# Efficacy, according to urodynamics, of OnabotulinumtoxinA compared with antimuscarinic drugs, for neurogenic detrusor overactivity: a systematic review and network meta-analysis

**DOI:** 10.1038/s41598-022-22765-1

**Published:** 2022-10-25

**Authors:** Rui Xu, Tong-Xin Yang, Ke-Wei Fang, Guang Wang, Pei Li

**Affiliations:** The Department of Urology, The Second Affiliated Hospital of Kunming Medical University, Kunming, Yunnan People’s Republic of China

**Keywords:** Urology, Urogenital diseases

## Abstract

To summarize the differences in urodynamic outcomes between oral antimuscarinic drugs and OnabotulinumtoxinA, and finding a therapy that maintains good urodynamics in neurogenic detrusor overactivity (NDO). We conducted a literature search of EMBASE and PubMed, with the language limited to English. In the analysis, all of the published randomized trials of OnabotulinumtoxinA or antimuscarinic drugs used to treat NDO were found and the results were finally obtained through Bayesian model analysis. A total of 12 RCTs and 2208 patients were included. OnabotulinumtoxinA 300U was superior to other drugs in terms of MCC, volume at IDC, and Pdet_max_ endpoints. OnabotulinumtoxinA 200U was more effective on the urodynamic endpoint of BC than other drugs or doses of OnabotulinumtoxinA. According to the MCC urodynamic results, oxybutynin, solifenacin 10 mg, and tolterodine 4 mg also had positive effects. OnabotulinumtoxinA 300U, 200U and 100U were better in improving the urodynamic results of NDO, and the current evidence also shows that selective injection of onabotulinumtoxinA can effectively improve the urodynamic results.

## Introduction

In 2002, the International Continence Society (ICS) defined neurogenic detrusor overactivity (NDO) as a relevant neurological condition, which is a urodynamic observation characterized by involuntary detrusor contractions during the filling phase that may be spontaneous or provoked^1^. NDO is a subset of neurogenic lower urinary tract dysfunction (NLUTD). Multiple sclerosis (MS) and Parkinson’s disease are common causes of NLUTD^2^. In prior terminology, “neurogenic bladder” was used to describe NLUTD’s condition. According to the AUA/SUFU, NDO’s treatments are related to surgical treatment, follow-up treatment, and non-surgical treatment^3^.

Oral antimuscarinic drugs are the first-line option for treating NLUTD. Previous research demonstrated that oral antimuscarinic drugs are effective in NDO and have a normal effect on increasing bladder capacity and lowering intravenous pressure^4,5^. OnabotulinumtoxinA is indicated for neurogenic detrusor overactivity. And it has been found that onabotulinumtoxinA has an impact on clinical and urodynamic results and helps to improve the quality of life in patients with neurogenic detrusor overactivity^6–8^.

In recent years, published studies aimed to evaluate the results of only using antimuscarinic drugs or OnabotulinumtoxinA for adults with neurogenic detrusor overactivity. Currently, there is a clear lack of evidence that has better efficacy in oral antimuscarinic drugs compared with OnabotulinumtoxinA in terms of urodynamic outcomes.

Comprehensive evaluations of the majority of meta-analysis studies indicate that OnabotulinumtoxinA is more effective than antimuscarinic drugs. Our study’s objective is to illustrate this issue from the standpoint of urodynamic testing indicators. Urodynamics are the primary indicators for determining the progression of neurogenic bladder disease. This study aimed to explain the differences between OnabotulinumtoxinA and antimuscarinic drugs using urodynamics. The objective of urodynamic evaluation is to figure a treatment to delay the progression of neurogenic bladder disease.

This study is the first network meta-analysis of randomized controlled trials (RCTs) using urodynamics results, maximum cystometric capacity (MCC), maximum detrusor pressure (Pdet_max_), volume at first involuntary detrusor contraction (IDC), and bladder compliance (BC) to assess the efficacy of oral antimuscarinic drugs compared with onabotulinumtoxinA. The study has been registered in PROSPERO (CRD42022328156).

## Patients and methods

### Retrieval strategy

According to the PICO framework^9^, the PICO is as follows: P-patients diagnosed with NDO according to the diagnostic criteria; I-interventions, including injection of OnabotulinumtoxinA and oral antimuscarinic drugs; C-comparison were placebo or different doses of OnabotulinumtoxinA; and O-using urodynamic outcomes, such as MCC, Pdet_max_, volume at first IDC, and BC. To identify suitable studies for network meta-analysis, two independent investigators conducted a comprehensive literature search of studies published in PubMed and EMBASE, with language restrictions limited to English. Follow the PRISMA extension recommendations checklist^10^. The search strategy used and associated synonyms are: “neurogenic bladder”, “neurogenic urinary bladder disorder”, “atonic neurogenic bladder”, “spastic neurogenic bladder”, “uninhibited neurogenic bladder” and “drug therapy”, “pharmacological therapy”, “solifenacin”, “tolterodine”, “oxybutynin”, “antimuscarinic therapy”, “Botulinum Toxin A”. Searches are performed using MeSH headings, free words, keywords, and combined searches using Boolean operators (OR and AND). (For detailed search strategies, please refer to Supplement Search strategies).

### Study inclusion/exclusion criteria

(i) Participants were adults; (ii) Studies were randomized controlled trials (RCTs); (iii) Urodynamic outcomes were included in the final outcome measure.

### Data extraction and assessment quality

Screening literature with software Endnote X9.1. Two investigators independently reviewed literature titles and abstracts for relevance and compliance with inclusion criteria. If the article's compliance is not clear from the abstract, the full text of the article is evaluated. The homogeneity of the included studies was also assessed.

Two investigators reviewed the quality of the included studies. The risk of bias was assessed using the RoB2 tool^11^. The tool evaluates the following: randomization process deviations from the intended interventions; missing outcome data; measurement of the outcome; and selection of the reported result. The quality of evidence was divided into three categories: low risk, some concerns, and high risk. Four urodynamic outcomes were: MCC, Pdet_max_, volume at first IDC, and BC. Those urodynamic outcomes were selected because they were associated with improved clinical symptoms. When standard deviation data was missing, the standard deviation was calculated from the data in the article or using the Cochrane handbook for systematic reviews of interventions.

### Data analysis

The network meta-analysis was performed using R software (R Core Team, R Foundation for Statistical Computing, Vienna, Austria, R version 4.1.1, 2021-08-10, https://www.R-project.org), STATA version 16.0 (StataCorp. 2019. Stata Statistical Software: Release 16. College Station, TX: StataCorp LLC), and OpenBUGS 3.2.3 (Andrew Thomas, 2014). All outcomes of interest were compared pairwise by calculating *I*^2^ statistics. Study heterogeneity was assessed using the R package. Node splitting analysis was performed to evaluate inconsistencies by comparing differences between direct and indirect evidence. Continuous variables were expressed as mean differences with 95% CIs, respectively. Using the RE model to calculate evidence inconsistencies and the ranking probabilities of different therapeutic drug interventions were also calculated.

## Results

### Included studies

**Figure 1:**
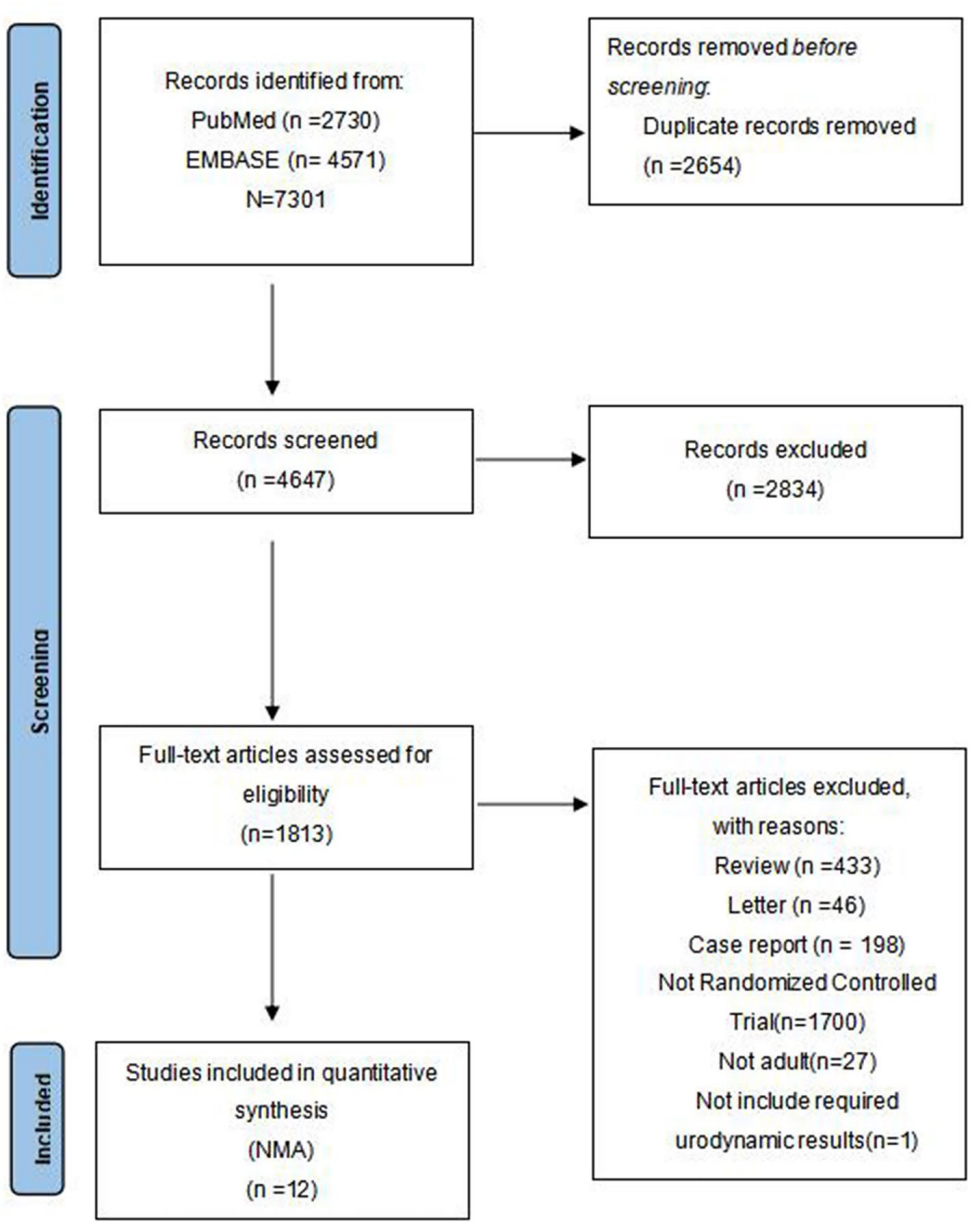
Preferred reporting items for systematic reviews and meta-analyses (PRISMA) flow diagram of the study selection process for network meta-analysis. The figure was generated using the PRISMA 2020 Flow Diagram.

Figure 1 depicts a flow diagram of the preferred reporting items for systematic reviews and meta-analyses (PRISMA) process of scoping literature. After searching through EMBASE and PubMed, the initial search discovered 4571 and 2730 expected research. The overall figure of papers was 7301 before duplicate removal. After screening, a total of 2834 papers were eliminated based on their abstract and/or title, whereas another 1800 papers were taken off after a full-text evaluation. An aggregate of 12 papers conformed to the qualitative inclusion standards, including 2208 participants, conformed to the standards for network meta-analysis and systematic review.

### Study and participant characteristics

The number of patients, the research design, the inventions for each of the involved research, and the outcomes are listed in Table 1. Table 2 summarizes study population characteristics and urodynamic outcomes as a result of the change from baseline.

**Table 1:** Baseline characteristics of included studies.

Author (year)[Ref.]	Study design, Country	Participants/lost to follow-up, n	Diagnoses	Group	Duration of treatment	Outcomes
Stöhrer (1991)^12^	RCT, Germany	61/6	detrusor hyperreflexia from SCI	Placebo	3-week	MCC, MDP, BC, Qmax, PVR
				Trospium chloride (20 mg Bid)		
Stöhrer (1999)^13^	RCT, Germany	113/11	detrusor hyperreflexia from SCI	Placebo	2-week	MCC, BC, PVR bladder capacity during onset of the first detrusor contraction, duration amplitude of the maximal detrusor contraction
				Propiverine (15 mg Tid)		
Stöhrer (2007)^14^	RCT, Germany	131/40	detrusor overactivity from SCI	Propiverine (15 mg Tid)	2-week	MCC, MDP, BC
				Oxybutynin (5 mg Tid)		
Madersbacher (1994)^15^	RCT, Austria	95/10	detrusor hyperreflexia from SCI	Trospium chloride (20 mg Bid)	2-week	MCC, MDP, BC, PVR
				Oxybutynin (5 mg Tid)		
Van Kerrebroeck (1998)^16^	RCT, Netherlands	90/14	detrusor hyperreflexia from SCI	Placebo	2-week	MCC, BC, PVR, Qmax, V_pmaxIDC_ volume at normal desire to void
				Tolterodine (0.5 mg Bid)		
				Tolterodine (1 mg Bid)		
				Tolterodine (2 mg Bid)		
				Tolterodine (4 mg Bid)		
Di Stasi (2001)^17^	RCT, Italy	12/0	detrusor hyperreflexia from SCI	Placebo	single dose	IDC, BC, PVR, UI episodes per week
				Oxybutynin (5 mg Tid)		
Cruz (2011)^18^	RCT, Canada	275/45	NDO from SCI or MS	Placebo	6-week	MCC, P_detmaxIDC_, V_pmaxIDC_, BC, UI episodes per week, incontinence quality of life, volume per void
				OnabotulinumtoxinA (200U)		
				OnabotulinumtoxinA (300U)		
Apostolidis (2012)^19^	RCT, Greece	74/45	NDO	Placebo	12-week	MCC, P_detmaxIDC_, V_pmaxIDC_, UI episodes per week, volume per void
				OnabotulinumtoxinA (50U)		
				OnabotulinumtoxinA (100U)		
				OnabotulinumtoxinA (200U)		
Ginsberg (2012)^20^	RCT, America	416/87	NDO from SCI or MS	Placebo	52-week	MCC, P_detmaxIDC_, 7-day bladder diary, UI episodes per week, incontinence quality of life
				OnabotulinumtoxinA (200U)		
				OnabotulinumtoxinA (300U)		
Rovner (2013)^21^	RCT, America	691/0	NDO from SCI or MS	Placebo	52-week	MCC, P_detmaxIDC_, V_pmaxIDC_, BC, percentage of patients with no IDC on post-treatment urodynamics at 6 weeks, 7-day bladder diary
				OnabotulinumtoxinA (200U)		
				OnabotulinumtoxinA (300U)		
Amarenco (2015)^22^	RCT, France	194/13	NDO from SCI or MS	Placebo	4-week	MCC, V_pmaxIDC_, MDP, micturition diary variables incontinence Quality of Life, Treatment Satisfaction Visual Analogue Scale, Euroqol 5-dimension questionnaire
				Oxybutynin (5 mg Tid)		
				Solifenacin (5 mg Qd)		
				Solifenacin (10 mg Qd)		
Ferreira (2018)^23^	RCT, Brazil	68/7	NDO from SCI	Oxybutynin (5 mg Tid)	24-week	MCC, P_detmaxIDC_, BC, incontinence episodes in 24 h, 3-day bladder diary
				OnabotulinumtoxinA (300U)		

*Bid* twice a day, *Tid* three times a day, *RCT* randomized controlled trial, *SCI* spinal cord injury, *MS* multiple sclerosis, *NDO* neurogenic detrusor overactivity, *IDC* first involuntary detrusor contraction, *MCC* maximum cystometric capacity, *BC* bladder compliance, *MDP* maximum detrusor pressure, *UI* urinary incontinence, *P*_*detmaxIDC*_ maximum detrusor pressure at first involuntary detrusor contraction, *V*_*pmaxIDC*_ volume at first IDC, *PVR* post-void residual, *Qmax* maximal urinary flow rate.

**Table 2:** Baseline population characteristics of included studies.

Author (year) [Ref.]	CG/EG, n	Mean age ± SD, years CG/EG	Male/female CG, EG	Urodynamics change from baseline Mean ± SD, CG/EG			
				MCC (ml)	Volume at first IDC (ml)	BC (cmH_2_O)	P_detmax_ (cmH_2_O)
Stöhrer (1991)^12^	PBO/TCL 28/27	(34.2 ± 10)/(32.3 ± 9.5)	(16/12), (14/13)	(2.5 ± 57.7)/(138.1 ± 107.5)	NM	(2.7 ± 4.5)/(12.1 ± 24.8)	(− 1.9 ± 11.8)/(− 37.8 ± 39)
Stöhrer (1999)^13^	PBO/Pro 53/60	(29.3 ± 10.9)/(30.3 ± 11.7)	(32/21), (37/23)	(− 7 ± 157.3)/(104 ± 137.8)	NM	(2 ± 11.4)/(5.2 ± 14.6)	(0.2 ± 42.4)/(− 27.1 ± 36.9)
Stöhrer (2007)^14^	Pro/Oxy 70/61	(38.8 ± 13.9)/(37.7 ± 15.1)	(54/16), (45/16)	(111 ± 122)/(134 ± 123)	NM	(11.9 ± 24.9)/(25.1 ± 47.5)	(− 19 ± 29.5)/(− 25.6 ± 36.7)
Madersbacher (1994)^15^	TCL/Oxy 52/43	(32.8 ± 10)/(31.3 ± 9)	(28/24), (19/24)	(96 ± 135.6)/(166 ± 137.4)	NM	(18.1 ± 24.7)/(22.6 ± 131.9)	(− 29 ± 33.1)/(− 38 ± 32.9)
Van Kerrebroeck (1998)^16^	PBO/Tol (0.5 mg) 19/20	(46 ± 15)/(39 ± 13)	(12/7), (15/5)	(43 ± 92)/(34 ± 116)	(40 ± 83)/(57 ± 108)	(8 ± 24)/(56 ± 65)	NM
	PBO/Tol (1 mg) 19/16	(46 ± 15)/(42 ± 15)	(12/7), (2/14)	(43 ± 92)/(82 ± 141)	(40 ± 83)/(54 ± 110)	(8 ± 24)/(− 11 ± 27)	
	PBO/Tol (2 mg) 19/18	(46 ± 15)/(40 ± 14)	(12/7), (8/10)	(43 ± 92)/(62 ± 160)	(40 ± 83)/(67 ± 100)	(8 ± 24)/(29 ± 37)	
	PBO/Tol (4 mg) 19/17	(46 ± 15)/(43 ± 14)	(12/7), (11/6)	(43 ± 92)/(154 ± 98)	(40 ± 83)/(136 ± 143)	(8 ± 24)/(22 ± 112)	
Di Stasi (2001)^17^	PBO/Oxy 12/12	NM	NM	NM	NM	(86.2 ± 65.8)/(103 ± 65.9)	NM
Cruz (2011)^18^	PBO/BTX-A (200U) 92/92	(46.9 ± 13.4)/(46 ± 13.1)	(43/49), (38/54)	(6.5 ± 144.8)/(157 ± 164.8)	(7.9 ± 141.2)/(182.7 ± 167.8)	(2.7 ± 97.8)/(71.5 ± 157.1)	(6.4 ± 41.1)/(− 28.5 ± 47.8)
	PBO/BTX-A (300U) 92/91	(46.9 ± 13.4)/(44.4 ± 13.9)	(43/49), (39/52)	(6.5 ± 144.8)/(157.2 ± 185.2)	(7.9 ± 141.2)/(199 ± 212.8)	(2.7 ± 97.8)/(60.9 ± 147.3)	(6.4 ± 41.1)/(− 26.9 ± 33.2)
Apostolidis (2012)^19^	PBO/BTX-A (50U) 16/19	(33.6 ± 9)/(31.2 ± 7.6)	(15/1), (17/2)	(117.4 ± 173.5)/(136.8 ± 189.4)	(110.8 ± 160.4)/(151.9 ± 176.1)	NM	(− 2.1 ± 27.7)/(− 20.1 ± 22.1)
	PBO/BTX-A (100U) 16/21	(33.6 ± 9)/(36.7 ± 11.7)	(15/1), (17/4)	(117.4 ± 173.5)/(220.1 ± 183.1)	(110.8 ± 160.4)/(215.4 ± 193)		(− 2.1 ± 27.7)/(− 29.4 ± 39.7)
	PBO/BTX-A (200U) 16/17	(33.6 ± 9)/(33.6 ± 10.8)	(15/1), (14/3)	(117.4 ± 173.5)/(183.7 ± 197.6)	(110.8 ± 160.4)/(243.4 ± 215.3)		(− 2.1 ± 27.7)/(− 33 ± 58.1)
Ginsberg (2012)^20^	PBO/BTX-A (200U) 149/135	(46 ± 13)/(46 ± 14)	(73/76), (55/80)	(16 ± 127)/(151 ± 171)	NM	NM	(− 2.4 ± 43.4)/(− 35.1 ± 35.7)
	PBO/BTX-A (300U) 149/132	(46 ± 13)/(47 ± 12)	(73/76), (43/89)	(16 ± 127)/(168 ± 170)			(− 2.4 ± 43.4)/(− 33.3 ± 37.8)
Rovner (2013)^21^	PBO/BTX-A (200U) 241/227	(46.2 ± 13.3)/(45.9 ± 13.3)	(116/125), (93/227)	(11.9 ± 134.3)/(153.6 ± 167.8)	(17.5 ± 133.2)/(183.4 ± 171.9)	(− 5.2 ± 116.3)/(59.8 ± 160.2)	(1.1 ± 42.6)/(− 32.4 ± 40.9)
	PBO/BTX-A (300U) 241/223	(46.2 ± 13.3)/(45.6 ± 13)	(116/125), (82/141)	(11.9 ± 134.3)/(163.1 ± 176.2)	(17.5 ± 133.2)/(202.4 ± 187.3)	(− 5.2 ± 116.3)/(50.4 ± 147.7)	(1.1 ± 42.6)/(− 30.1 ± 35.4)
Amarenco (2015)^22^	PBO/Oxy 43/47	(40 ± 10.6)/(43.9 ± 11.9)	(23/20), (19/28)	(5.4 ± 120.3)/(165.4 ± 145.6)	(− 10.1 ± 83.1)/(113.4 ± 101.4)	NM	(7.5 ± 51.0)/(− 24.3 ± 27.6)
	PBO/Sol (5 mg) 43/48	(40 ± 10.6)/(44.6 ± 12.5)	(23/20), (27/21)	(5.4 ± 120.3)/(77.8 ± 115.4)	(− 10.1 ± 83.1)/(60 ± 109.2)		(7.5 ± 51.0)/(− 16.6 ± 32.9)
	PBO/Sol (10 mg) 43/51	(40 ± 10.6)/(45.7 ± 12)	(23/20), (26/25)	(5.4 ± 120.3)/(134.2 ± 124.7)	(− 10.1 ± 83.1)/(79.2 ± 122.3)		(7.5 ± 51.0)/(− 10.5 ± 37.2)
Ferreira (2018)^23^	Oxy/BTX-A (300U) 34/34	(31 ± 8)/(33 ± 11)	(26/7), (23/5)	(126 ± 62)/(289 ± 135)	NM	(7 ± 5)/(26 ± 24)	(− 21 ± 20)/(− 49 ± 29)

*CG* control group, *EG* experimental group, *NM* not mentioned, *MCC* maximum cystometric capacity, *IDC* first involuntary detrusor contraction, *BC* bladder compliance, *Pdet*_*max*_ maximum detrusor pressure, *PBO* placebo, *TCL* trospium chloride, *Pro* propiverine, *Oxy* oxybutynin, *Tol* tolterodine, *BTX-A* onabotulinumtoxinA, *Sol* solifenacin.

### Networks

**Figure 2:**
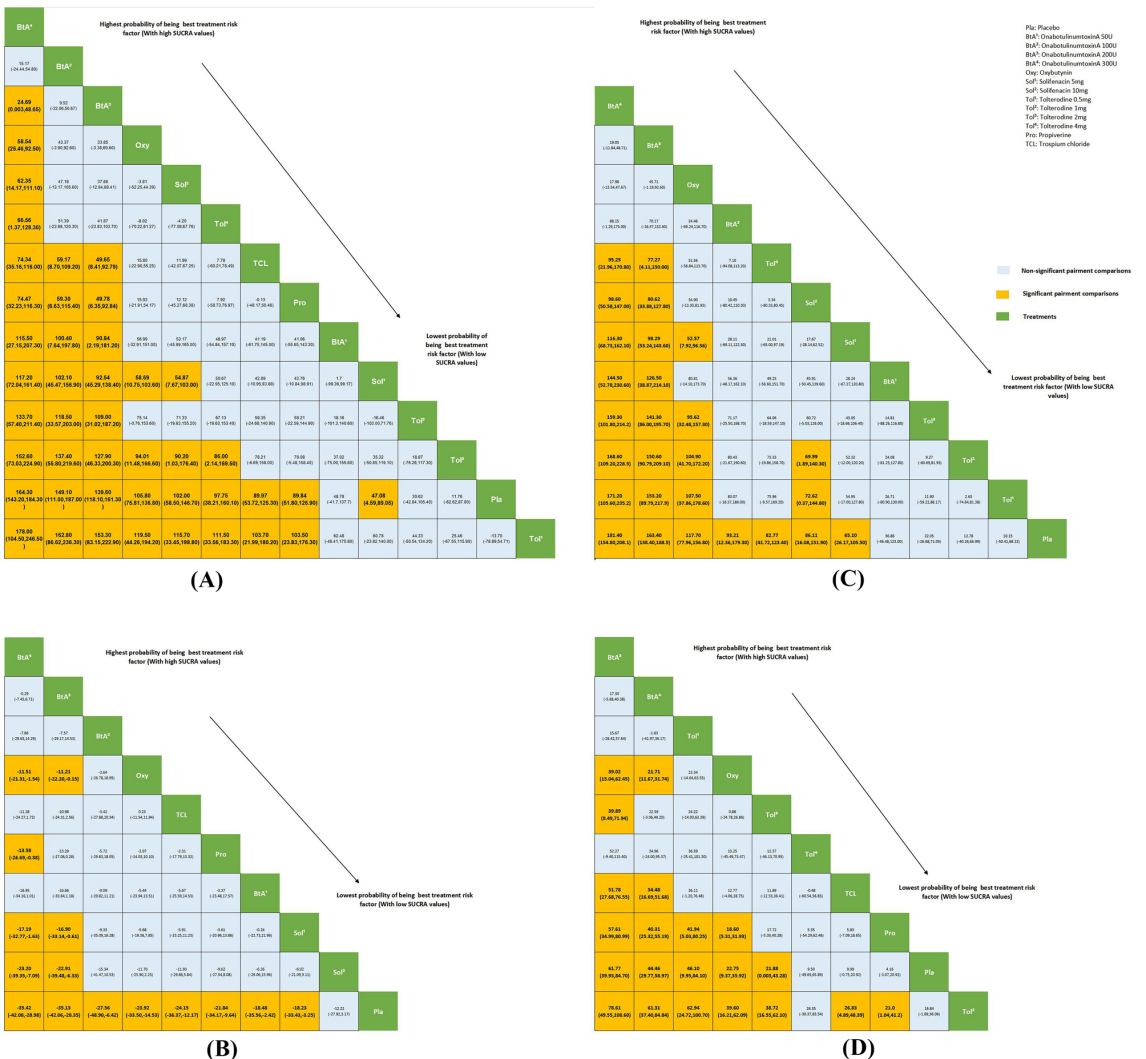
Pairwise comparison based on urodynamic results according to network meta-analysis. Treatments are orders in the rank of their chance of being the best treatment. The green boxes are treatments, the orange boxes are significant pairment comparisons, the cornflower blue boxes are non-significant pairment comparisons (the numbers are mean and the credible intervals). (**A**) MCC comparison of multiple treatments. (**B**) Pdet_max_ comparison of multiple treatments. (**C**) Vol at First IDC comparison of multiple treatments. (**D**) Bladder compliance comparison of multiple treatments. *Pla* Placebo, *BtA*^*1*^ OnabotulinumtoxinA 50U, *BtA*^*2*^ OnabotulinumtoxinA 100U, *BtA*^*3*^ OnabotulinumtoxinA 200U, *BtA*^*4*^ OnabotulinumtoxinA 300U, *Oxy* Oxybutynin, *Sol*^*1*^ Solifenacin 5 mg, *Sol*^*2*^ Solifenacin 10 mg, *Tol*^*1*^ Tolterodine 0.5 mg, *Tol*^*2*^ Tolterodine 1 mg, *Tol*^*3*^ Tolterodine 2 mg, *Tol*^*4*^ Tolterodine 4 mg, *Pro* Propiverine, *TCL* Trospium chloride. Non-significant pairment comparisons, Significant pairment comparisons, Treatments.

There was a deficiency of adequate evidence to make comparisons, and the pair-wise comparison meta-analysis outcomes are demonstrated in Fig. 2.

### Risk of bias

**Figure 3:**
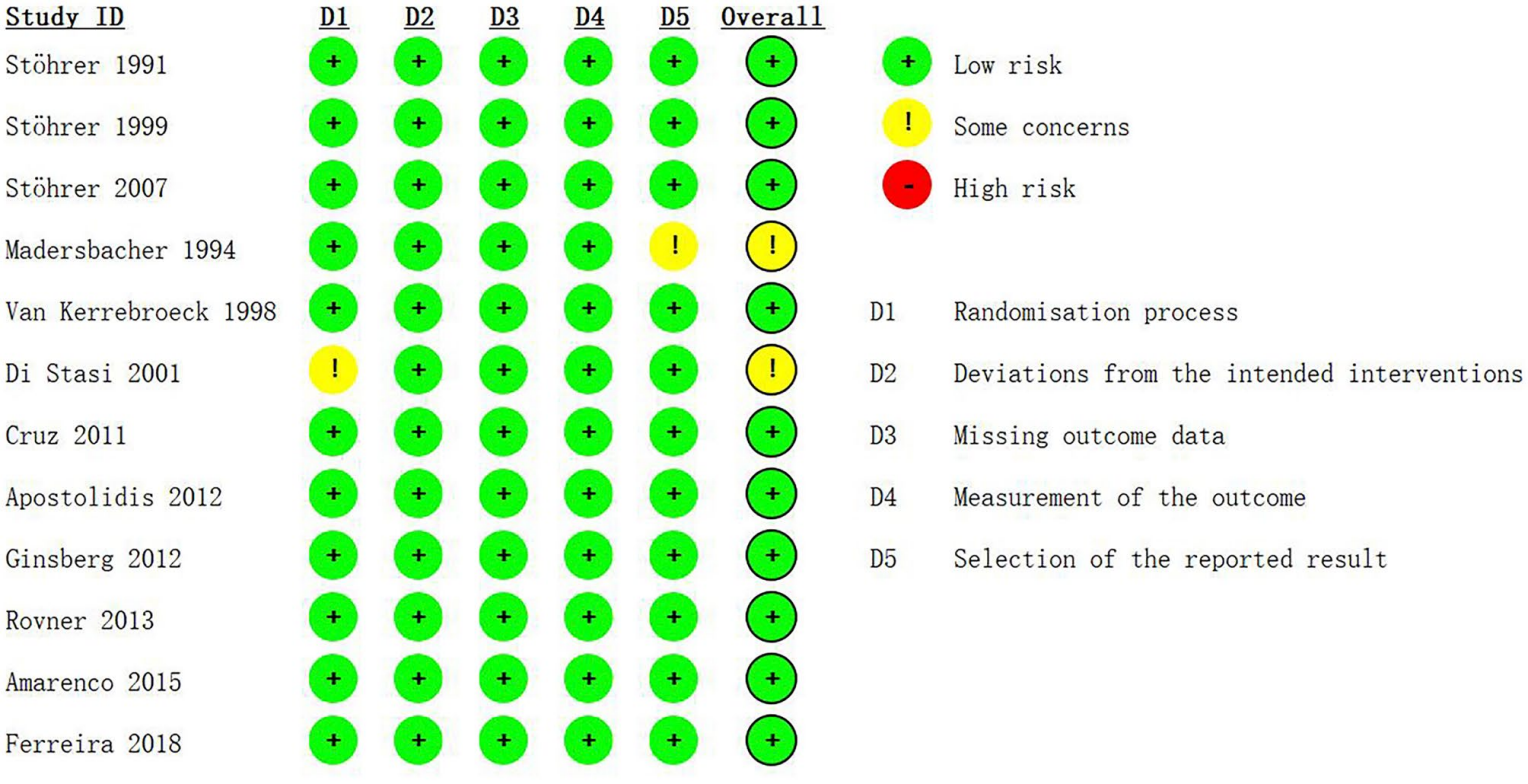
Risk of bias figure using ROB 2 tool. D1: Randomisation process; D2: deviations from the intended interventions; D3: missing outcome data; D4: measurement of outcome; D5: selection of the reported result. Low risk, Some concerns, High risk.

The risk of bias figure is shown in Fig. 3, using the ROB 2 tool (the 22nd August 2019 version).

### Efficacy

#### Maximum cystometric capacity (MCC)

A total of 11 studies (n = 2196) contributed to the NMA (Network Meta-analysis) results. Pair-wise comparisons using a random effects (RE) model show that 300U onabotulinumtoxinA injection has a statistically significant difference in efficacy compared to almost all drugs (Fig. 2A). There was no statistical significance among the three doses of onabotulinumtoxinA (300U, 100U, and 200U). Compared with onabotulinumtoxinA 300U, the credible interval (*CI*) of onabotulinumtoxinA 100U is (− 24.44, 54.80), which is not statistically significant. According to SUCRA values, the top three therapeutic drugs were: onabotulinumtoxinA 300U, onabotulinumtoxinA 100U, and onabotulinumtoxinA 200U.

#### Maximum detrusor pressure (Pdet_max_)

Ten studies (n = 2106) reported maximum detrusor pressure (Pdet_max_) date. solifenacin 10 mg [*CI*: (− 27.92, 3.17)] has no significant differences compared with placebo. According to SUCRA values, the top three therapeutic drugs were: onabotulinumtoxinA 300U, onabotulinumtoxinA 200U, and onabotulinumtoxinA 100U. (Fig. 2B).

#### Volume at first involuntary detrusor contraction (IDC)

According to five studies (n = 1318), final findings remain that onabotulinumtoxinA 200U compared to onabotulinumtoxinA 300U [*CI*: (− 11.84, 48.71)] is not statistically significant. The top three in the ranking of SUCRA values were: onabotulinumtoxinA 300U, onabotulinumtoxinA 200U, and oxybutynin (Fig. 2C).

#### Bladder compliance (BC)

Nine studies (n = 1255) that contributed to the results show that tolterodine 2 mg [*CI*: (− 46.13, 70.95)] has no significant differences compared with tolterodine 4 mg. The ranking according to SUCRA values is onabotulinumtoxinA 200U, onabotulinumtoxinA 300U, and tolterodine 0.5 mg (Fig. 2D).

## Discussion

Urodynamic testing is the most objective method to identify abnormalities of the bladder and urethra during the filling and storage phase during the voiding phase of neurogenic bladder dysfunction. And assessing the outcomes of interventions is one of the five main indications for the use of urodynamic studies^24,25^.

The definition of maximum bladder capacity varies. In our meta-analysis, the definition we used was the maximum intravesical instillation that patients could withstand when awake for urodynamic testing^26^. This indicator can reflect the maximum urine storage capacity of the bladder. The larger the capacity, the better the urine storage function of the patient might be and the more suitable for clean intermittent catheterization (CIC). However, since the factor of bladder pressure is not included, cases with high maximum bladder capacity may not necessarily have mild symptoms and may not necessarily have no ureteral reflux.

From the results, almost all drugs can increase the MCC after increasing the dose, indicating that all drugs can achieve a certain degree of relaxation of the muscle tension of the bladder wall; onabotulinumtoxinA 300U, 100U, and 200U have the best effects, followed by oxybutynin, solifenacin 10 mg, and tolterodine 4 mg, and finally, trospium chloride and propiverine were also statistically significant. However, since bladder pressure was not taken into account, whether this difference was clinically meaningful needs to be combined with other indicators.

Pdet_max_ refers to the maximum registered detrusor pressure during voiding^26^. Pdet_max_ is inversely related to renal function; the greater the maximal detrusor pressure, the worse the renal function^27,28^. There are two conditions of maximum pressure, one is the case of detrusor hyperreflexia during urine storage, which is defined as the pressure when the detrusor muscle contracts to the highest peak, reflecting the severity of detrusor hyperreflexia. The second is the absence of detrusor hyperreflexia during storage but the presence of a low compliance bladder. The intravesical pressure is the maximum pressure when the maximum bladder capacity is reached, reflecting the severity of the low compliance bladder. These two conditions are not easy to distinguish in literature. However, researchers usually use the maximum bladder pressure during storage to reflect the severity of detrusor hyperreflexia.

In our study, all drugs except solifenacin 10 mg could reduce detrusor pressure. This suggests that patients who have lower urinary tract symptoms and urge incontinence after treatment can get relief. Upon review of the original study, solifenacin 10 mg was found to be statistically significant when compared with placebo. However, the lack of statistical significance in NMA may be due to the low number of cases. It is worth noting that the effect of a 300U onabotulinumtoxinA injection is the best, at least not worse than 200U. In other words, 300U of onabotulinumtoxinA injection can achieve the best effect of relieving detrusor hyperreflexia. Meanwhile, oral drug therapy was effective for detrusor hyperreflexia, which had a worse effect on detrusor hyperreflexia than 300U onabotulinumtoxinA, and most of the oral drug differences were statistically significant. In summary, 300U of onabotulinumtoxinA injection could be the first choice in clinical practice.

During the filling phase, involuntary detrusor contractions may occur^29^. NDO is a urodynamic observation characterized by IDC. Volume at IDC takes into account both bladder capacity and bladder function during storage. There are three conditions after treatment: a. The bladder capacity of the primary detrusor uninhibited contraction did not change, but the contraction pressure did; b. the bladder capacity of the primary detrusor uninhibited contraction increased, but the contraction pressure remained unchanged; c. the bladder capacity increased while the contraction pressure decreased during the detrusor's primary uninhibited contraction. All three conditions can be considered to have improved; this indicator only shows the difference in the latter two conditions.

Our findings remain that all drugs at a given dose can increase volume at first involuntary detrusor contraction (IDC), and onabotulinumtoxinA injections were more effective than oral drug therapy. The 300U onabotulinumtoxinA injection had the best effect. Thus, 300U of onabotulinumtoxinA may have had a better effect on delayed detrusor muscle contraction without inhibition and reducing detrusor muscle contraction pressure. As for oral antimuscarinic drugs, they were not as effective as onabotulinumtoxinA and were similar in efficacy to each other.

The result of bladder compliance is the most important indicator, which takes into account both bladder capacity and intravesical pressure, and is a key indicator for the progression of neurogenic bladder disease. Regarding bladder compliance, the following points should be noted: a. Bladder fibrosis and bladder contracture are changes in the end-stage neurogenic bladder. Elastin in the extracellular matrix of the bladder wall is associated with compliance^30^. The fibrotic bladder loses extensibility, and bladder pressure increases significantly when filling a small amount. The lower the compliance, the more severe the bladder fibrosis; b. Bladder compliance derives from the concept of safe bladder capacity, which refers to the bladder capacity when the intravesical pressure reaches 40 cm H_2_O. Neurogenic bladders are poorly compliant and may develop persistent detrusor hypertension. Detrusor leak pressure or maximum bladder filling pressure greater than 40 cm H_2_O increases the risk of upper urinary tract injury^25,31^. c. The efficacy of drug therapy in improving bladder compliance may also be related to the compliance of the enrolled patients. d. Bladder compliance does not consider the problem of detrusor hyperreflexia, which is evaluated by two indicators: maximum detrusor pressure and bladder capacity during initial uninhibited detrusor contraction.

According to our final results, maintaining good urodynamic parameters is facilitated more by the 200U onabotulinumtoxinA protocol; tolterodine 4 mg was ineffective in improving bladder compliance. Oxybutynin had the effect of improving bladder compliance. In the absence of onabotulinumtoxinA injection, oxybutynin drug treatment is better for maintaining good urodynamic indicators. About the dose of 0.5 mg tolterodine, *Van Kerrebroeck *et al*.*^16^ *“*There was no apparent dose–response relationship for the effect of tolterodine on the number of bladder compliance”. In summary, under the conditions of onabotulinumtoxinA injection, the 200U onabotulinumtoxinA injection treatment plan has more advantages for maintaining good urodynamic indices.

It is worth mentioning that onabotulinumtoxinA injection is a minimally invasive procedure for the neurogenic bladder and is used when there is no response to drug therapy and the bladder wall is not fully fibrotic. Urodynamic testing indices are essential for determining the progression of neurogenic bladder disease. This study aims to explain the difference between onabotulinumtoxinA injection and antimuscarinic drugs using urodynamic indices.

It should be noted that when patients were initially included in the study, no special difference was made for their inclusion criteria. Included in the inclusion criteria were both NDO patients who had received ineffective drug treatment and NDO patients who had not been treated with drugs. In the initial study, the final efficacy endpoint did not differentiate between two patient groups. In addition, the cost of treatment, side effects, and patient satisfaction of onabotulinumtoxinA injection were not investigated. Therefore, in follow-up studies, comprehensive comparisons can be made to finally find a suitable treatment plan for the individual patient.

## Conclusion

This meta-analysis showed that onabotulinumtoxinA injection was more effective for the treatment of NDO. None of the different oral antimuscarinic drugs or different doses evaluated in this review were superior to onabotulinumtoxinA injection. Accoding to the urodynamic indicators, onabotulinumtoxinA injection into the bladder wall be implemented earlier and even become a necessary treatment for NDO, rather than being considered only when oral drug therapy fails. Since this review did not compare the adverse effects of treatment, the incidence of adverse reactions between oral antimuscarinic drugs and onabotulinumtoxinA is not known.

## Supplementary Information

Supplementary Information.

## Data Availability

The datasets generated during and/or analysed during the current study are available from the corresponding author on reasonable request.

## Abbreviations

NDO: Neurogenic detrusor overactivity
NLUTD: Neurogenic lower urinary tract dysfunction
SCI: Spinal cord injury
MS: Multiple sclerosis
RCTs: Randomized controlled trails
MCC: Maximum cystometric capacity
Pdet_max_: Maximum detrusor pressure
IDC: First involuntary detrusor contraction
BC: Bladder compliance
CIC: Clean intermittent catheterization
*CI*: Credible interval
