# Supplementary material for: Efficacy, according to urodynamics, of OnabotulinumtoxinA compared with antimuscarinic drugs, for neurogenic detrusor overactivity: a systematic review and network meta-analysis
Source: Sci Rep. 2022 Oct 25;12:17905. doi: 10.1038/s41598-022-22765-1 (PMC9606369; doi:10.1038/s41598-022-22765-1)
Supplement: Supplementary file 1 — Supplementary Information. [file 41598_2022_22765_MOESM1_ESM.pdf]

Pubmed Search strategy

Using ***PubMed Advanced Search Builder,*** choosing “**All Fields**” in ***Add terms to the query box***. The followings are ***Query box*** and ***Search Details***.

#1 ： Search : (((((((neurogenic bladder) OR (neurogenic urinary bladder)) OR (neuropathic bladder)) OR (neurogenic urinary bladder disorder)) OR (neurogenic bladder disorder)) OR (atonic neurogenic bladder)) OR (spastic neurogenic bladder)) OR (uninhibited neurogenic bladder))

#2 ： Search : (((((((((((drug) OR (drug therapy)) OR (drug treatment)) OR (pharmacological therapy)) OR (tolterodine tartrate)) OR (solifenacin succinate)) OR (solifenacin)) OR (tolterodine)) OR (oxybutynin)) OR (antimuscarinic agents)) OR (antimuscarinic therapy)) OR (Botulinum Toxin A)

#3 ： Search: ((((((((neurogenic bladder) OR (neurogenic urinary bladder)) OR (neuropathic bladder)) OR (neurogenic urinary bladder disorder)) OR (neurogenic bladder disorder)) OR (atonic neurogenic bladder)) OR (spastic neurogenic bladder)) OR (uninhibited neurogenic bladder)) AND ((((((((((((drug) OR (drug therapy)) OR (drug treatment)) OR (pharmacological therapy)) OR (tolterodine tartrate)) OR (solifenacin succinate)) OR (solifenacin)) OR (tolterodine)) OR (oxybutynin)) OR (antimuscarinic agents)) OR (antimuscarinic therapy)) OR (Botulinum Toxin A))

Embase Search strategy

#1 'neurogenic bladder'

#2 'neurogenic urinary bladder'

#3 'neuropathic bladder'

#4 'neurogenic urinary bladder disorder'

#5 'neurogenic bladder disorder'

#6 'atonic neurogenic bladder'

#7 'spastic neurogenic bladder'

#8 'uninhibited neurogenic bladder'

#9 drug

#10 'drug therapy'

#11 'drug treatment'

#12 'pharmacological therapy'

#13 'tolterodine tartrate'

#14 'solifenacin succinate'

#15 solifenacin

#16 tolterodine

#17 oxybutynin

#18 'antimuscarinic agents'

#19 'antimuscarinic therapy'

#20 'botulinum toxin a'

#21 #1 OR #2 OR #3 OR #4 OR #5 OR #6 OR #7 OR #8

#22 #9 OR #10 #11 OR #12 OR #13 OR #14 OR #15 OR #16 OR #17 OR #18 OR #19 OR #20

#23 #21 AND #22
